# Metabolic engineering of tomato fruit enriched in L-DOPA

**DOI:** 10.1016/j.ymben.2020.11.011

**Published:** 2021-05

**Authors:** Dario Breitel, Paul Brett, Saleh Alseekh, Alisdair R. Fernie, Eugenio Butelli, Cathie Martin

**Affiliations:** a Department of Metabolic Biology and Biological Chemistry, The John Innes Centre, Norwich Research Park, Norwich, NR4 7UH, UK; b Max-Planck-Institut Fur Molekulare Pflanzenphysiologie, Am Muhlenberg 1, 14476, Potsdam-Golm, Germany; c Tropic Biosciences, Innovation Centre, Norwich Research Park, NR4 7GJ, UK

**Keywords:** Tomato, L-DOPA, MYB12, Shelf-life, Melanin

## Abstract

L-DOPA, also known as Levodopa or L-3,4-dihydroxyphenylalanine, is a non-standard amino acid, and the gold standard drug for the treatment for Parkinson's Disease (PD). Recently, a gene encoding the enzyme that is responsible for its synthesis, as a precursor of the coloured pigment group betalains, was identified in beetroot, *BvCYP76AD6*. We have engineered tomato fruit enriched in L-DOPA through overexpression of *BvCYP76AD6* in a fruit specific manner. Analysis of the transgenic fruit revealed the feasibility of accumulating L-DOPA in a non-naturally betalain-producing plant. Fruit accumulating L-DOPA also showed major effects on the fruit metabolome. Some of these changes included elevation of amino acids levels, changes in the levels of intermediates of the TCA and glycolysis pathways and reductions in the levels of phenolic compounds and nitrogen-containing specialised metabolites. Furthermore, we were able to increase the L-DOPA levels further by elevating the expression of the metabolic master regulator, *MYB12*, specifically in tomato fruit, together with *BvCYP76AD6*. Our study elucidated new roles for L-DOPA in plants, because it impacted fruit quality parameters including antioxidant capacity and firmness. The L-DOPA levels achieved in tomato fruit were comparable to the levels in other non-seed organs of L-DOPA - accumulating plants, offering an opportunity to develop new biological sources of L-DOPA by widening the repertoire of L-DOPA-accumulating plants. These tomato fruit could be used as an alternative source of this important pharmaceutical.

## Highlights

• Tomato fruit were engineered to synthesise and accumulate L-DOPA.
• Co-expression of the transcription factor, MYB12, doubled the levels of L-DOPA in tomato fruit.
• The accumulation of L-DOPA resulted in additional changes in the profile of primary and secondary metabolites in tomatoes.
• The L-DOPA tomato fruit exhibited improved shelf life and reduced susceptibility to *Botrytis cinerea*.

## 1 Introduction

L-DOPA, also known as Levodopa or L-3,4-dihydroxyphenylalanine, has been the gold standard therapy for Parkinson's Disease (PD) since its establishment as a drug in 1967 (Cilia et al., 2017; Hornykiewicz, 2010). It is one of the essential medicines, as declared by the World Health Organisation (WHO Model List, Essential Medicines, 19th edition, April 2015). The market volume of L-DOPA was 101 billion dollars and 250 tons per year, in 2005 (Koyanagi et al., 2005). The most common source of L-DOPA is chemical synthesis but biological and natural sources are also available (Patil et al., 2013). Only a few plants have been reported to contain measurable quantities of L-DOPA, mainly in seeds, with the most studied and best known being the velvet bean, *Mucuna pruriens,* which contains up to 10% w/w L-DOPA in its seeds (Ramya and Thaakur, 2007).

Although L-DOPA draws a lot of attention as a drug, its role in plants has not been extensively investigated. It was suggested to have repellent properties in preventing seeds from being attacked, or defensive roles in velvet bean (Rehr et al., 1973). It can also serve as an allelochemical to prevent neighbouring plants from growing nearby, once excreted from the roots (Fujii et al., 1991). The toxicity of L-DOPA results in inhibition of root growth and has been attributed to the fact that L-DOPA is a precursor for melanin and causes damage while it is being polymerised (Hachinohe and Matsumoto, 2005). The toxic effects of L-DOPA can be reversed by decreasing the activity of Polyphenol Oxidase (PPO), that promotes its oxidation, or by application of ascorbic acid (Soares et al., 2014; Hachinohe and Matsumoto, 2007). In high concentrations, L-DOPA has been shown to have strong antioxidant properties, as well (Sivanandhan et al., 2015; Gülçin, 2007). L-DOPA is an essential precursor for synthesis of betalain pigments and for some specialised alkaloids, such as epinephrine and codeine (Polturak and Aharoni, 2018).

**Fig. 1:**
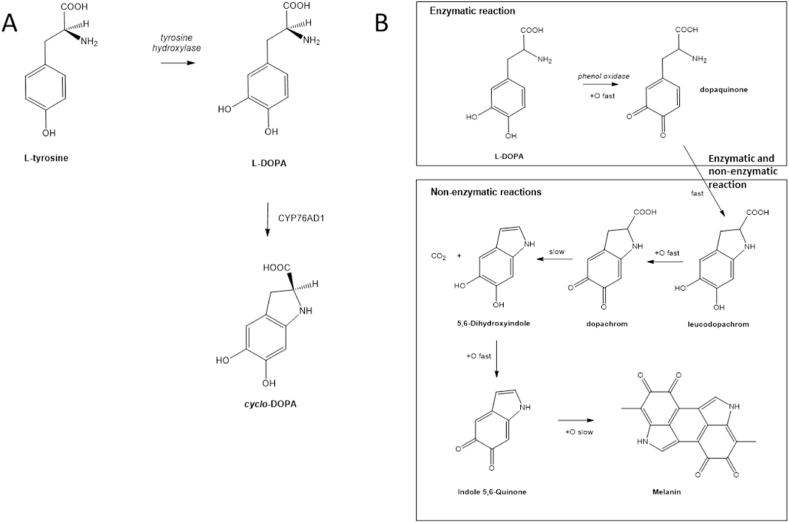
L-DOPA Biosynthetic pathway (A) L-DOPA is synthesised through hydroxylation of tyrosine. Further hydroxylation by CYP76AD1, in beetroot, for example, results in *cyclo*-DOPA. (B) Melanin formation is initiated through enzymatic oxidation of L-DOPA to dopaquinone. This is followed by enzymatic and non-enzymatic oxidation reactions that result in the formation of leucodopachrome that is further oxidised to melanin, via several non-enzymatic steps.

In several organisms, such as walnut and the fungus *Lentinula edodes*, L-DOPA is synthesised through the hydroxylation of L-tyrosine by tyrosinases which are PPOs (Sullivan, 2014) (Fig. 1). Recently, progress in understanding the biosynthesis of betalains was made, when a group of CYP450 proteins was identified in beetroot (*Beta vulgaris*) that catalyse the conversion of tyrosine to L-DOPA, in the first step of the betalain biosynthetic pathway (Sunnadeniya et al., 2016). While some of these CYP450 enzymes, such as the beetroot CYP76AD1, have dual activity in tyrosine hydroxylation and oxidation of L-DOPA to cyclodopa, the very closely related protein, CYP76AD6 catalyses only tyrosine hydroxylation (Polturak et al., 2016). To date, no metabolic engineering of a non-betalain synthesising plant to accumulate L-DOPA has been reported.

L-DOPA is naturally produced in some plants, and in certain circumstances it is administered from natural plant sources (Cummings, 1991; Barbeau, 1969). We wanted to explore the feasibility of engineering its synthesis in plants that do not normally accumulate it, and to offer opportunities for new phyto-production of L-DOPA. Tomato is a crop model with fleshy fruit and a complete well-characterised genome, platforms for comprehensive analysis of gene expression, extensive metabolite analysis data, useful genetic resources and a wide range of analytical tools and protocols (Fei et al., 2011; Klee and Giovannoni, 2011; Tomato Genome, 2012). These tools and resources, together with tomato being one of the most commonly-consumed crops in the world with its high nutritional value in the human diet, have made tomato a preferred chasis for metabolic engineering (Raiola et al., 2014; Butelli et al., 2008). In this study, L-DOPA-accumulating tomatoes were generated, in a fruit-specific manner, to avoid yield penalties or possible toxicity effects of L-DOPA on plant development (Fujii et al., 1991). Tomato fruit were the organ of choice, since they are relatively rich in ascorbate, which could prevent the oxidation of L-DOPA and the generation of melanin, which might, in turn, cause further oxidative stress (Fig. 1B) (Soares et al., 2014; Stevens et al., 2007). We report the effects of ectopic L-DOPA production in this crop on metabolite and fruit quality properties, and the feasibility of synthesis of L-DOPA for development of new biological sources.

## 2 Materials and methods

### 2.1 Yeast transformation

*BvCYP76AD6* and *BvCYP76AD1* were cloned in pAG423GAL-ccdB and pYES-DEST52 as previously described (Polturak et al., 2016), and transformed into BY4742 yeast strain using the polyethylene glycol/lithium acetate (PEG/LiAc) method. Vectors transformed with *BETA-GLUCORONIDASE* (*GUS*) instead of the CYP76 genes, were used as controls. Yeast were grown in synthetic defined (SD) medium overnight, containing 2% glucose and lacking amino acids as necessary for selection. The yeast were pelleted and resuspended to OD_600_=1 in SD medium with 2 mM ascorbate, 1% raffinose and 2% galactose and lacking the relevant amino acids. Cultures were sampled (500 μl) as detailed in the main text, freeze-dried and analysed for L-DOPA levels, using 1 ml of extraction buffer.

### 2.2 Generation of CYP76AD6 tomato plants

pBIN-E8-BVCYP76AD6 was cloned using pDONR207-BvCYP76AD6 (Polturak et al., 2016) and pJIT160-E8 (Luo et al., 2008) using Gateway recombination according the the manufacturer's instructions (Invitrogen). *Agrobacterium tumefaciens*-mediated transformation of tomato cv. Money Maker was carried out as previously described (Tian et al., 2007). Transgenic plants were confirmed by kanamycin resistance and PCR amplification using gene- and promoter-specific primers.

### 2.3 Extracts of tomato fruit

Fruit were harvested seven days post breaker, with at least three independent, biological replicates and placenta and seeds were removed, before freezing in liquid nitrogen. Samples were stored at −80 °C until ground in liquid nitrogen and analysed.

### 2.4 Gene expression analysis

RNA was extracted from fruit with Tri Reagent, according to the manufacturer's protocol (Sigma), following lithium acetate precipitation. DNaseI-treated RNA samples (Sigma), were reverse transcribed using a High Capacity cDNA reverse transcription kit (Applied Biosystems). Gene expression levels were analysed using SYBR® Green JumpStart™ Taq ReadyMix™ (Sigma) and BioRad CFX reaL-time PCR instruments. *TIP41* was used as an endogenous control (Exposito-Rodriguez et al., 2008).

### 2.5 L-DOPA measurements

Fruit was extracted for 30 min shaking at room temperature in extraction buffer (80% MeOH, 0.1% formic acid), with 50ug ml^−1^ labelled L-DOPA [L-Dopa-(phenyl-d3)] as internal standard (cat. #333786, Sigma). This was followed by 5 min sonication, centrifugation at 4 °C and filtration of the supernatant through a 0.22 μm filter. Extracts were diluted 1000 times in 0.1% formic acid. Samples were kept at 4 °C in the dark until injected. Standard curves were generated for 0–50 μg ml^−1^ L-DOPA (Sigma).

Samples were analysed using Waters Acquity LC combined with a Xevo TQS mass spectrometer and Accucore-150-Amide-HILIC 2.6u 100*2.1 mm column. The mobile phase was 0.1% formic acid/acetonitrile 20/80 changing to 30/70 over 4 min at a flow rate of 0.4 ml min^−1^. L-DOPA eluted at 2.3 min and was identified in positive ion mode by a m/z 152.06 fragment which was normalised to the 154.33 mass of the labelled L-DOPA.

### 2.6 Firmness and water loss analysis

Fruit were harvested 14 days post breaker and stored at 16 °C, in the dark, in a nylon bag. The fruit were weighed and scored for firmness (1–5 scale; 5-hardest, 1-softest) every week, for five weeks.

### 2.7 Antioxidant capacity analysis, GC-MS and LC-MS for metabolite analyses

For metabolic analyses, ground samples were freeze-dried overnight and extracted (30 mg ml^−1^) in extraction buffer (ribitol 1.5 mg l^−1^ in 80% methanol). Samples were shaken at room temperature for 30 min, followed by 10 min sonication and 10 min centrifugation in 4 °C.

For the Trolox equivalent antioxidant capacity assays (TEAC), 5 μl of the supernatant were used for the analysis, as previously described (Pellegrini et al., 2003).

For GC-MS, the ribitol-methanol extract was derivitized for 90 min at 37 °C (in 50 μl of 20 mg ml^−1^ methoxyamine hydrochloride in pyridine) followed by a 30 min treatment at 37 °C with 120 μl of MSTFA (*N*-Methyl-*N*-(trimethylsilyl)trifluoroacetamide, *N*-Trimethylsilyl-*N*-methyl trifluoroacetamide). The GC-MS system used was a gas chromatograph coupled to a time-of-flight mass spectrometer (Leco Pegasus HT TOF-MS). A Gerstel Multi Purpose autosampler system injected the samples. Helium was used as carrier gas at a constant flow rate of 2 ml s^−1^ and gas chromatography was performed on a 30 m DB-35 column. The injection temperature was 230 °C and the transfer line and ion source were set to 250 °C. The initial temperature of the oven (85 °C) increased at a rate of 15 °C min^−1^ up to a final temperature of 360 °C. After a solvent delay of 180 s, mass spectra were recorded at 20 scans s^−1^ with m/z 70–600 scanning range. Chromatograms and mass spectra were evaluated by using Chroma TOF 4.5 (Leco) and TagFinder 4.2 software(Schauer et al., 2008).

Secondary metabolites were profiled by the Waters Acquity UPLC system coupled to the Q-Exactive Orbitrap mass detector according to the previously published protocol(Giavalisco et al., 2009). The UPLC system was equipped with a HSS T3 C18 reversed phase column (100 × 2.1 mm i.d., 1.8-μm particle size; Waters) that was operated at a temperature of 40 °C. The mobile phases consisted of 0.1% formic acid in water (Solvent A) and 0.1% formic acid in acetonitrile (Solvent B). The flow rate of the mobile phase was 400 μl min^−1^, and 3 μl of sample was loaded per injection. The UPLC was connected to an Exactive Orbitrap (Thermo Fisher Scientific) via a heated electrospray source (Thermo Fisher Scientific). The spectra were recorded using full scan mode for negative ion detection, covering a mass range from m/z 100 to 1500. The resolution was set to 25,000, and the maximum scan time was set to 250 ms. The sheath gas was set to a value of 60, while the auxiliary gas was set to 35. The transfer capillary temperature was set to 150 °C, while the heater temperature was adjusted to 300 °C. The spray voltage was fixed at 3 kV, with a capillary voltage and a skimmer voltage of 25 and 15 V, respectively. MS spectra were recorded from minute 0 to 19 of the UPLC gradient. Molecular masses, retention time, and associated peak intensities were extracted from the raw files using RefinerMS (version 5.3; GeneData), and Xcalibur software (Thermo Fisher Scientific) (Alseekh et al., 2015).

Metabolite identification and annotation of the GC-MS and UPLC runs were performed using standard compounds, literature, and tomato metabolomics databases (Alseekh et al., 2015; Moco et al., 2006; Iijima et al., 2008; Tohge and Fernie, 2009). Values were obtained as relative to the internal standards, ribitol and isovitexin for GC-MS and UPLC-MS, respectively.

### 2.8 *Botrytis cinerea* wound inoculation

*Botrytis cinerea* wound inoculations and scoring in tomato fruit were carried out as previously described (Bassolino et al., 2013).

### 2.9 Melanin extraction

The method of extraction of melanin was modified from one previously described (Zhu and Chen, 2009). For the analysis of intact tissue, fruit were harvested at 14 days post anthesis (dpa), and treated as previously described. For analysis of melanin levels in injured fruit, a horizontal cut was made using a scalpel on fruit at 7 dpa, while on the vine. Seven days later, fruit were harvested, and tissues from 0.5 cm around the cut were dissected and processed. The tissues were ground and freeze dried for analyses. Thirty mg tissue was treated with 750 μl hydrochloride acid (6M) for 5 h at room temperature, followed by 30 min centrifugation at 14,000 rpm. Pellets were then extracted with 600 μl sodium hydroxide for 20 min at 70°, followed by 5 min centrifugation. Supernatants were added to 300 μl of 6M hydrochloric acid and centrifuged for 30 min. Pellets were washed twice with water, resuspended in 10 mM NaOH, and incubated at 37 °C for an hour. The melanin levels were determined by measuring the absorbance at 410 nm.

### 2.10 Oligonucleotides and accession numbers used in this study

All oligonucleotides and accession numbers used in this study are detailed in Refer DIB article.

## 3 Results

### 3.1 CYP76AD6 vs CYP76AD1 activity in yeast

**Fig. 2:**
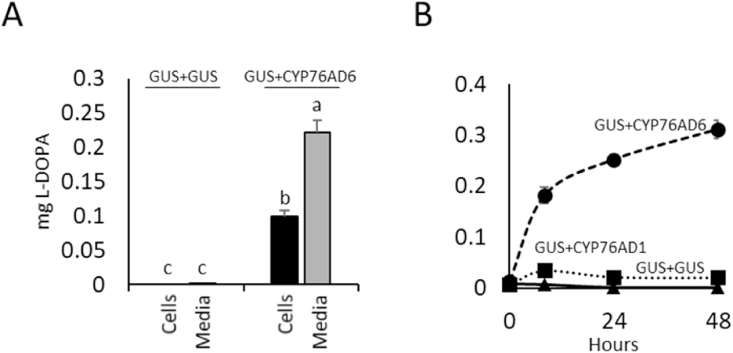
Comparison of the activity of *CYP76AD1* and *CYP76AD6* in yeast harbouring expression cassettes of *BvCYP76AD1* or *BvCYP76AD6* were analysed for L-DOPA accumulation levels, compared to a control cassette expressing the *GUS* gene. (A) L-DOPA was released into the growth medium, although a large amount of the metabolite remained inside the yeast cells. (B) Although *BvCYP76AD6* and *BvCYP76AD1* could both drive the synthesis of L-DOPA in yeast, *BvCYP76AD6* was much more efficient.

Recently, two genes were found to catalyse the hydroxylation of tyrosine to L-DOPA in beetroot, *CYP76AD1* and *CYP76AD6* (Polturak et al., 2016). To decide which gene should be used to generate L-DOPA-enriched tomato fruit, we took advantage of the established BY4742 yeast system (Polturak et al., 2016) (Fig. 2). First, we checked whether L-DOPA accumulated in the yeast cells or was released into the medium. Yeast transformed with *CYP76AD6*, under the GAL1 inducible promoter were induced for 72 h. The L-DOPA levels were quantified separately in the medium and in the pelleted yeast cells (Fig. 2A). There was a significant difference between the fractions, and the medium contained twice the amount of L-DOPA as the yeast fraction. Therefore, we decided to measure the L-DOPA levels in cells and medium together, to determine total L-DOPA production.

To compare the ability of CYP76AD1 and CYP76AD6 to produce L-DOPA, yeast cells expressing *CYP76AD1* or *CYP76AD6* were generated and analysed (Fig. 2B). A construct harbouring a *GUS* expression cassette was used as a control. L-DOPA levels were monitored up to 48 h post induction and normalised to an OD_600_ of 1. L-DOPA accumulation in yeast strains expressing the *CYP76AD1* was much lower than the levels accumulating in strains expressing *CYP76AD6*. *CYP76AD1* reached saturation after 8 h, and still produced about five times lower levels of L-DOPA than *CYP76AD6*-expressing yeast at the same time. After 48 h, the L-DOPA levels in the *CYP76AD6*-expressing yeast were 14 times higher than in the *CYP76AD1*-expressing lines.

These yeast expression assays suggested that CYP76AD6 would promote the accumulation of L-DOPA in plants better than CYP76AD1, and therefore it was chosen for engineering L-DOPA accumulation in tomatoes.

### 3.2 Generation of L-DOPA - engineered tomato plants

Since L-DOPA has been shown to have toxic effects and might inhibit growth leading to yield losses, we made use of the fruit specific E8 promoter of tomato, to drive the overexpression of *CYP76AD6* gene (Hachinohe and Matsumoto, 2007; Butelli et al., 2008; Luo et al., 2008; Itkin et al., 2009). The *CYP76AD6* gene from beetroot was cloned into an expression cassette containing the E8 promoter and 35S terminator from CaMV. Three stably transformed tomato lines were established, containing the *CYP76AD6* expression cassette (CYP76AD6-#1, CYP76AD6-#2, CYP76AD6-#3). Fruit from these plants were analysed seven days post breaker (Br+7) for *CYP76AD6* gene expression levels, by quantitative Reverse Transcription PCR (qRT-PCR), with samples from wild type (wt) fruit as controls (Table 1). All three transformed lines expressed the *CYP76AD6* transgene, however line #3 showed lower expression of *CYP76AD6* than line #1 and line #2. We completed this analysis by quantifying the L-DOPA levels in these fruit (Table 1). Fruit from CYP76AD6-#3, in line with the lower expression of *CYP76AD6*, contained only 0.002% L-DOPA (2.38 mg per 100g fruit fresh weight), while lines #1 and #2 contained ~ 0.01% each (CYP76AD6-#1 had 10.43 mg and CYP76AD6-#2 had 8.48 mg per 100g fruit), while wt fruit showed only traces of L-DOPA. Since biological sources of L-DOPA have been reported, mainly in seeds (Ramya and Thaakur, 2007) which, unlike tomatoes, have low water content, we freeze dried the fruit tissue to estimate L-DOPA levels on the basis of dry weight. Once water had been eliminated from the tissue (91–95%) the L-DOPA content was 0.04% in CYP76AD6-#3, 0.18% in CYP76AD6-#1 and 0.13% in CYP76AD6-#2 of the total dry weight of the fruit. Wt L-DOPA levels in this analysis were 5.5 × 10^−5^% of the fruit dry weight, which was judged to be effectively zero.

**Table 1:** **Gene expression analysis and L-DOPA accumulation in fruit from T1 plants:** Fruit from three lines of CYP76AD6 transgenic plants were harvested seven days post breaker and analysed for *CYP76AD6* expression, and L-DOPA accumulation in fresh tissue and in dry tissue. As expected, the *BvCYP76AD6* transgene was expressed only in the transgenic lines CYP786AD6#1-#3. These fruit accumulated L-DOPA at a higher level than wt fruit, which contained only trace amounts of L-DOPA. Significant changes, compared to wt, were identified using a Student's t-test, ^a^- P-value<0.0001; ^b^-P-value<0.005; ^c^- P-value<0.05.

	Gene Expression		L-DOPA - Per 100g Fresh Weight		L-DOPA - per 100g Dry Weight	
	-dCt	stdev	mg/100g	stdev	mg/100g	stdev
wt	−13.39	±1.48	0.03	±0.04	0.005	±0.001
CYP76AD6-#1	2.50	±3.08^a^	10.43	±1.68 ^a^	1.85	±0.69 ^b^
CYP76AD6-#2	2.30	±0.11 ^a^	8.47	±1.63 ^a^	1.33	±0.34 ^b^
CYP76AD6-#3	0.43	±1.47 ^a^	2.38	±1.98	0.39	±0.29 ^c^

These results showed that the heterologous expression of *BvCYP76AD6* for the synthesis of L-DOPA was not only feasible, but these plants were also capable of storing L-DOPA, in a sink organ (fruit). This motivated us to investigate the effects of accumulating L-DOPA *in vivo*, further. We carried out more detailed analyses of lines #1 and #2, which had the highest levels of L-DOPA among the analysed lines.

### 3.3 Changes in the metabolic profile in L-DOPA containing fruit

**Fig. 3:**
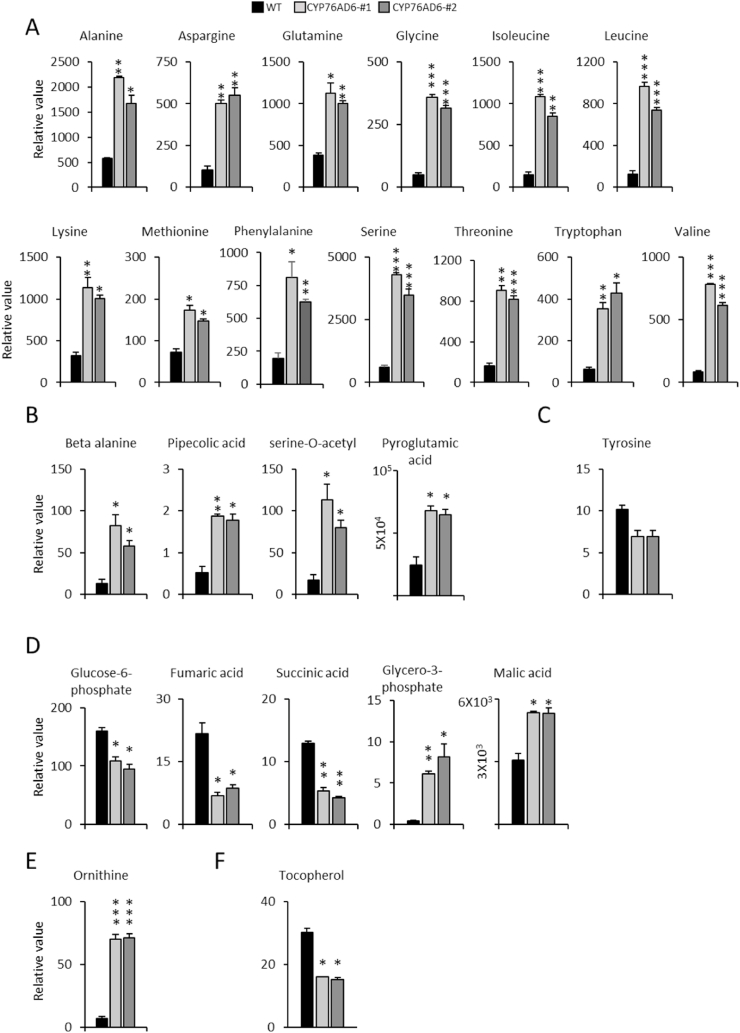
**Changes in the profile of primary metabolites in L-DOPA-enriched tomatoes:** GC-MS analysis of fruit expressing *CYP76AD6* harvested seven days post breaker was carried out to identify any changes in the profile of primary metabolites. Fruit with high levels of L-DOPA showed significantly higher levels of (A) standard and (B) non-standard amino acids. (C) Tyrosine levels were reduced in the fruit enriched in L-DOPA, but values were not statistically significantly lower than the control. (D) Intermediates in glycolysis, the TCA cycle and the Calvin cycle, showed altered levels. (E) L-DOPA-enriched fruit had higher levels of the polyamine ornithine, and (F) reduced levels of tocopherol. Student's t-test significance compared to WT: (*) P-value<0.05; (**) P-value<0.01; (***) P-value<0.001.

**Fig. 4:**
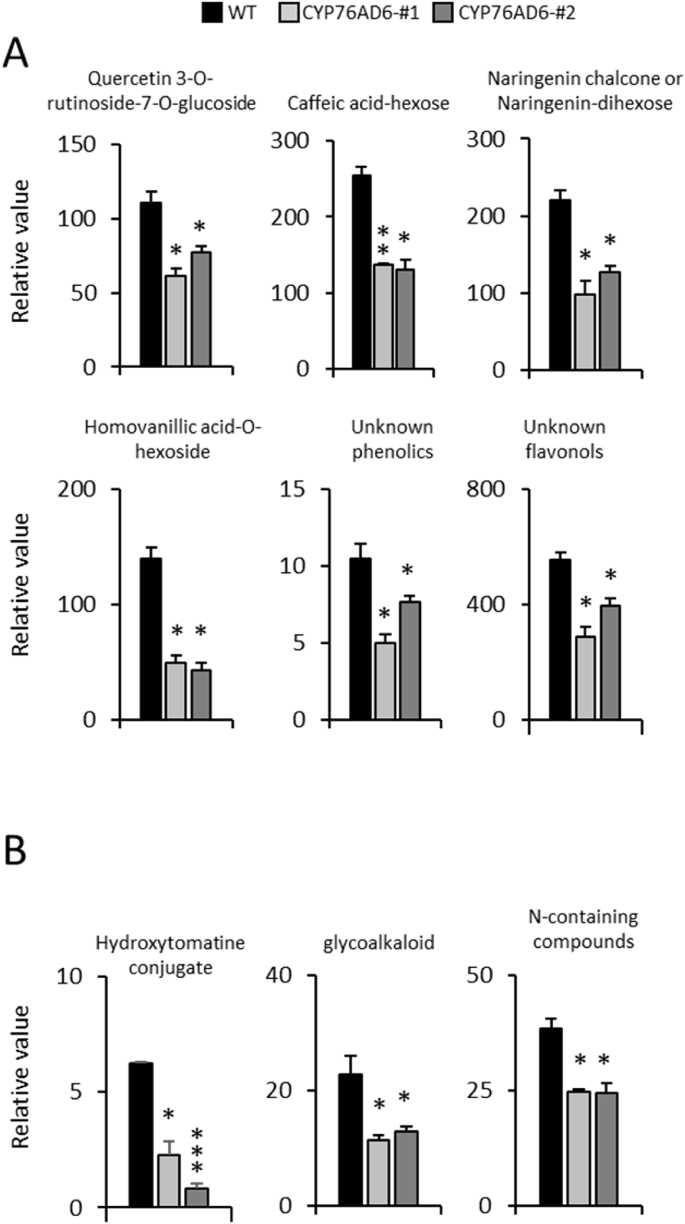
**Changes in the profiles of secondary metabolites in L-DOPA-enriched tomatoes:** LC-MS analysis of L-DOPA-enriched tomato fruit, harvested seven days post breaker, was carried out to identify changes in the profiles of secondary metabolites. Fruit with high levels of L-DOPA showed reduced levels of (A) polyphenols and (B) nitrogen-containing compounds (alkaloids). Student's t-test significance compared to WT- (*) P-value<0.05; (**) P-value<0.01; (***) P-value<0.001.

L-DOPA is a secondary metabolite that is synthesised from tyrosine, a primary amino acid, the synthesis of which is tightly regulated (Sullivan, 2014). We explored the effect of L-DOPA synthesis and accumulation on the metabolite profiles of tomato fruit. To do this, we carried out untargeted metabolite analysis by GC-MS (Fig. 3; refer DIB article) and LC-MS (Fig. 4; Refer DIB article) on fruit, 7 days post-breaker, from lines CYP76AD6-#1 and CYP76AD6-#2.

Changes in primary metabolites were identified primarily by GC-MS analysis (Fig. 3; refer DIB article). We observed higher levels of most amino acids in L-DOPA -accumulating fruit than in wt fruit, including significantly higher levels of alanine, asparagine, glutamine, glycine, isoleucine, leucine, lysine, methionine, phenylalanine, serine, threonine, tryptophan, and valine (Fig. 3A). In addition, nonstandard amino acids and other downstream metabolites derived from standard amino acids increased in L-DOPA fruit (Fig. 3B). These metabolites included beta alanine, pipecolic acid, serine-O-acetyl and pyroglutamic acid, which has been suggested to function as a storage form of glutamate (Kumar and Bachhawat, 2012). Tyrosine levels were reduced compared to wt fruit, which was not surprising since tyrosine is consumed in the biosynthesis of L-DOPA. However, these reductions in tyrosine levels were not statistically significant (Fig. 3C), emphasising the likely tight regulation of tyrosine levels. Levels of other compounds annotated as intermediates in glycolysis, the TCA cycle and the Calvin cycle also showed alterations. The L-DOPA accumulating fruit had lower levels of glucose-6-phosphate, fumaric acid and succinic acid, and higher levels of glyceroL-3-phosphate and malic acid (Fig. 3D). Interestingly, the levels of the polyamine ornithine were 10 times higher in the L-DOPA fruit than in wt fruit (Fig. 3E). Tocopherol levels were reduced by 50% in the transgenic fruit. Tocopherols require tyrosine as an intermediate in their biosynthesis, suggesting that available tyrosine was redirected for the synthesis of L-DOPA, instead of other tyrosine-dependant metabolites in CYP76AD6 fruit (Fig. 3F).

Further investigation of the metabolome in L-DOPA-accumulating fruit, primarily for secondary metabolites, was carried out by LC-MS (Fig. 4; Refer DIB article). Identification was based on m/z ionization signals. Unlike the changes that were observed in primary metabolites and their derivatives by GC-MS, all the specialised metabolites that exhibited changes, were reduced compared to wt (Fig. 4). Two groups of specialised metabolites were reduced significantly, polyphenols (Fig. 4A) and nitrogen containing compounds (Fig. 4B). Among the reduced polyphenols were quercetin 3-O-rutinoside-7-O-glucoside, caffeic acid-hexose, naringenin chalcone, naringenin-dihexose, homovanillic acid-O-hexoside, and other phenolics and flavonols that were not identified, specifically. All these polyphenols are derived from phenylalanine via general phenylpropanoid biosynthesis, suggesting that tyrosine consumption for L-DOPA production feeds back to repress general phenylpropanoid metabolism. Since phenylalanine levels increased in CYP76AD6 fruit, this implies that this feedback control operates on the activity of PAL (phenylalanine ammonia lyase) as reported for L-DOPA treatment of soyabean ] (Bido et al., 2018). Among the nitrogen containing compounds, conjugates of glycoalkaloids (including hydroxytomatine) and other N-containing compounds, showed reduced levels in fruit accumulating L-DOPA.

Our analyses suggested that the consumption of tyrosine to form L-DOPA induced primary metabolism to form higher levels of most amino acids, while reducing the synthesis of specialised metabolites that compete for aromatic amino acids in tomato fruit.

### 3.4 Overexpression of *CYP76AD6* in the background of *MYB12* results in further accumulation of L-DOPA

**Fig. 5:**
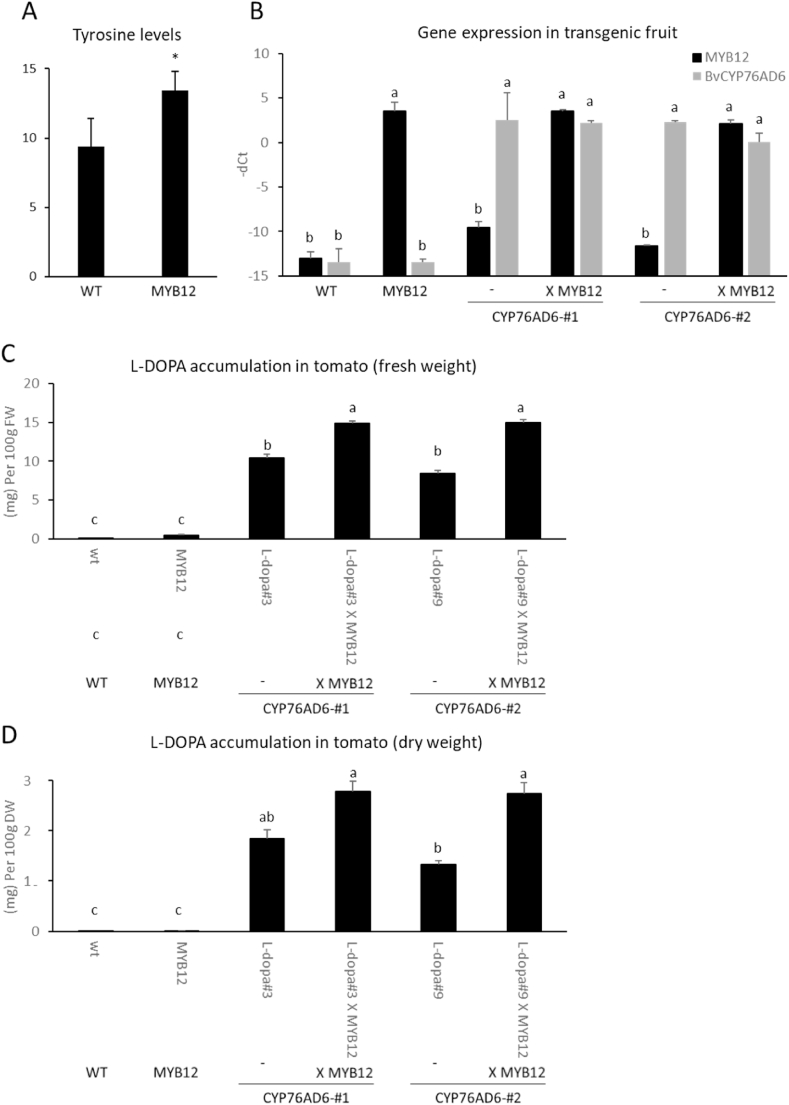
**Analysis of L-DOPA levels in tomato fruit expressing*****CYP76AD6:*** (A) Tyrosine levels in Breaker + seven days (Br+7) fruit, from *MYB12*- overexpressing plants were analysed by GC-MS and found to be higher than in the wt fruit. (B–D) Fruit from *CYP76AD6*-expressing plants and their crosses with MYB12 plants were harvested seven days post breaker and analysed for (A) transgene expression, (C) L-DOPA accumulation in fresh tissue and (D) in dry tissue. (A) The *BvCYP76AD6* transgene was expressed only in the transgenic lines CYP786AD6#1-#2 and their crosses with MYB12. *MYB12* was expressed only in the MYB12 line and in its crosses to CYP76AD6. (B,C) L-DOPA accumulated in the *CYP76AD6*-expressing tomato lines. The L-DOPA levels increased when *MYB12* overexpression was also introduced to the plants. Student's t-test significance: P-value<0.05.

Zhang et al (2015) reported that overexpression of Arabidopsis *MYB12* in tomato fruit results in higher levels of tryptophan and tyrosine, the precursor of L-DOPA compared to the wt (Zhang et al., 2015). Seeds of a characterised, homozygous MYB12 line in the MoneyMaker genetic background (Luo et al., 2008) were grown to maturity. Tomato fruit from plants confirmed to be overexpressing Arabidopsis *MYB12* (MYB12), were harvested at 7 days post breaker, and analysed by GC-MS. Tyrosine levels were ~50% higher than in wt fruit, confirming the previous findings (Fig. 5A). Therefore, to increase the L-DOPA accumulation in the CYP76AD6 fruit further, two independent lines (CYP76AD6-#1, CYP76AD6-#2) were crossed with tomatoes overexpressing the *MYB12* gene from Arabidopsis (MYB12) specifically in fruit (Luo et al, 2008). Fruit harvested from F1 plants, 7 days post breaker (Br+7), were analysed for gene expression, together with wild type (wt) and MYB12 fruit as controls, and fruit harvested from the T2 plants of the CYP76AD6 lines (Fig. 5B). The two original L-DOPA accumulating lines expressed the *CYP76AD6* transgene, as expected, and the expression levels of the *CYP76AD6* and *MYB12* transgenes were not significantly different between the crosses (*CYP76AD6*#1XMYB12 and *CYP76AD6*#2XMYB12 lines) and their *CYP76AD6* and MYB12 parents.

Next, we analysed the L-DOPA levels in this set of fruit (Fig. 5C). The lines crossed with MYB12 showed increased L-DOPA accumulation, to 0.015% of the fruit fresh weight (14.9 mg/100g fruit), which was 30–45% more than in the CYP76AD6 parental line. In addition, in freeze-dried fruit, the CYP76AD6XMYB12 crossed fruit contained on average 0.27% L-DOPA on the basis of dry weight (Fig. 5D). These results were in line with increased flux of carbon from primary metabolism to its precursor, tyrosine, caused by *MYB12* expression in fruit (Tian et al., 2007).

The colour of MYB12 tomatoes changes to yellow/orange principally as a result of the high levels of pale yellow flavonols produced in fruit (Luo et al., 2008). No changes in total carotenoid levels were observed in these fruit(Luo et al., 2008). The fruit of CYP76AD6XMYB12 crosses were also yellow/orange in appearance and LC/MS confirmed that fruit of these crosses also accumulated high levels of flavonols (Refer DIB article).

### 3.5 Accumulation of L-DOPA enhances fruit quality properties

**Fig. 6:**
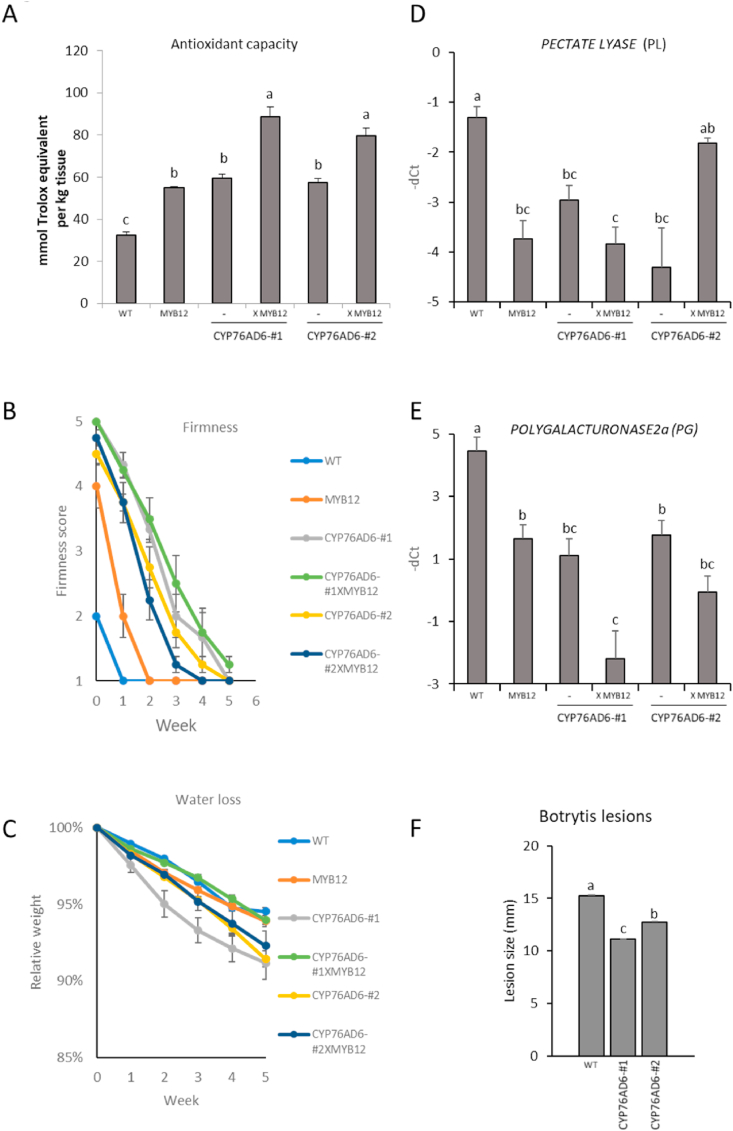
**Fruit shelf life analyses in L-DOPA-enriched tomatoes:** Parameters correlated with fruit shelf life were studied in L-DOPA accumulating tomatoes and in fruit from crosses with MYB12. Fruit with higher contents of L-DOPA exhibited (A) higher antioxidant capacity, and (B) kept firm for longer after harvest. The firmness of the fruit was correlated with low expression levels of (D) *PECTATE LYASE* and (E) *POLYGALACTURONASE*. (C) No significant differences were observed in the water loss rate between the different genotypes. (F) In addition, L-DOPA accumulating fruit exhibited smaller lesions than wt following inoculation with *Botrytis cinerea*. Student's t-test significance- P-value<0.05.

L-DOPA has been reported to have strong antioxidant properties, established using several different assays (Gülçin, 2007; Aware et al., 2017). It has been estimated to have a relative TEAC (Trolox Equivalent Antioxidant Capacity) value of 1.92 whereas its precursor, tyrosine had a value of 0.04 (Dimic et al., 2019). Therefore we tested the L-DOPA-accumulating tomatoes for their antioxidant capacities using the Trolox assays (Fig. 6A) (Pellegrini et al., 2003). Fruit from both CYP76AD6-#1 and #2 lines, showed higher antioxidant capacities than wt fruit. Increases in antioxidant capacities in MYB12 tomato fruit relative to wt fruit have already been reported (Pandey et al., 2015). Similar antioxidant capacities to those previously reported were observed for MYB12 fruit (Fig. 6A) (Luo et al., 2008). Interestingly, fruit from CYP76AD6 lines crossed with MYB12, had higher antioxidant capacities than either of the parental lines individually, confirming the positive association between L-DOPA levels and antioxidant capacity.

Higher levels of antioxidants and improved antioxidant capacities have been shown to be positively associated with improved shelf life in many soft fruits (Zhang et al., 2013; Park et al., 2015; Andrade-Cuvi, 2017; Jayachandran et al., 2007). To test whether the high antioxidant capacity of tomato fruit enriched in L-DOPA impacted shelf-life as well, we recorded the changes in fruit firmness and water loss in detached fruit (Fig. 6B and C), from fourteen days post-breaker to five weeks later. The rate of water loss was slightly faster in the L-DOPA-accumulating fruit than in wt fruit, although these differences were not statistically significant. In contrast, starting from the point of harvest, the rate of fruit softening was dramatically different. Fruit from wt plants were already soft at fourteen days post breaker, completing softening within a week. Upon harvest, MYB12 and the L-DOPA fruit were firmer than wt fruit. The MYB12 fruit were slightly less firm than the *CYP76AD6*-expressing fruit. The differences in firmness between the genotypes were more obvious one week post-harvest; MYB12 fruit underwent faster softening, reaching their softest points earlier than the L-DOPA-accumulating lines. Fruit expressing *CYP76AD6* showed complete softening three to five weeks post-harvest. Interestingly, fruit from both CYP76AD6XMYB12 crosses showed no significant difference in firmness compared to their respective L-DOPA-accumulating parental lines, suggesting that there were no additive effects of *MYB12* overexpression beyond the effects caused by L-DOPA-accumulation on fruit shelf-life. Softening of tomato fruit during ripening is correlated with the expression levels of two genes, *PECTATE LYASE* (*PL*) and *POLYGALACTURONASE2a* (PG) (Mintz-Oron et al., 2008; Uluisik et al., 2016). We analysed the expression of these softening-associated genes by qRT-PCR in the transgenic fruits, seven days post breaker (Fig. 6D and E). Expression of both, *PL* and *PG*, was reduced in all fruit accumulating L-DOPA as well as in fruit expressing *MYB12*, in line with the reduced softening rates observed in these fruits, compared to wt. The expression levels of these two markers of over-ripening were not significantly different in the L-DOPA fruit from those in MYB12 fruit. Since the L-DOPA fruit showed delayed softening compared to the MYB12 fruit, it may be that L-DOPA contributes to fruit firmness in additional ways.

A second feature important to shelf life of tomato is the response of fruit to infection by pathogens. We analysed the effect of L-DOPA accumulation on the response of tomato fruit to the necrotrophic fungus, *Botrytis cinerea*. Fruit at 14–21 days post-breaker, were wounded and inoculated with the pathogen and lesions were examined three days post inoculation (dpi). While fungal inoculation of wt fruit caused the development of mycelium, fruit accumulating L-DOPA showed no signs of mycelium and had smaller lesions (Fig. 6F). These results strengthened further the association between antioxidant capacity, fruit firmness upon ripening and susceptibility to pathogens, as previously reported in tomato fruit and other soft fruit crops (Zhang et al., 2013; Polturak et al., 2017).

### 3.6 L-DOPA-accumulating tomatoes produce melanin

**Fig. 7:**
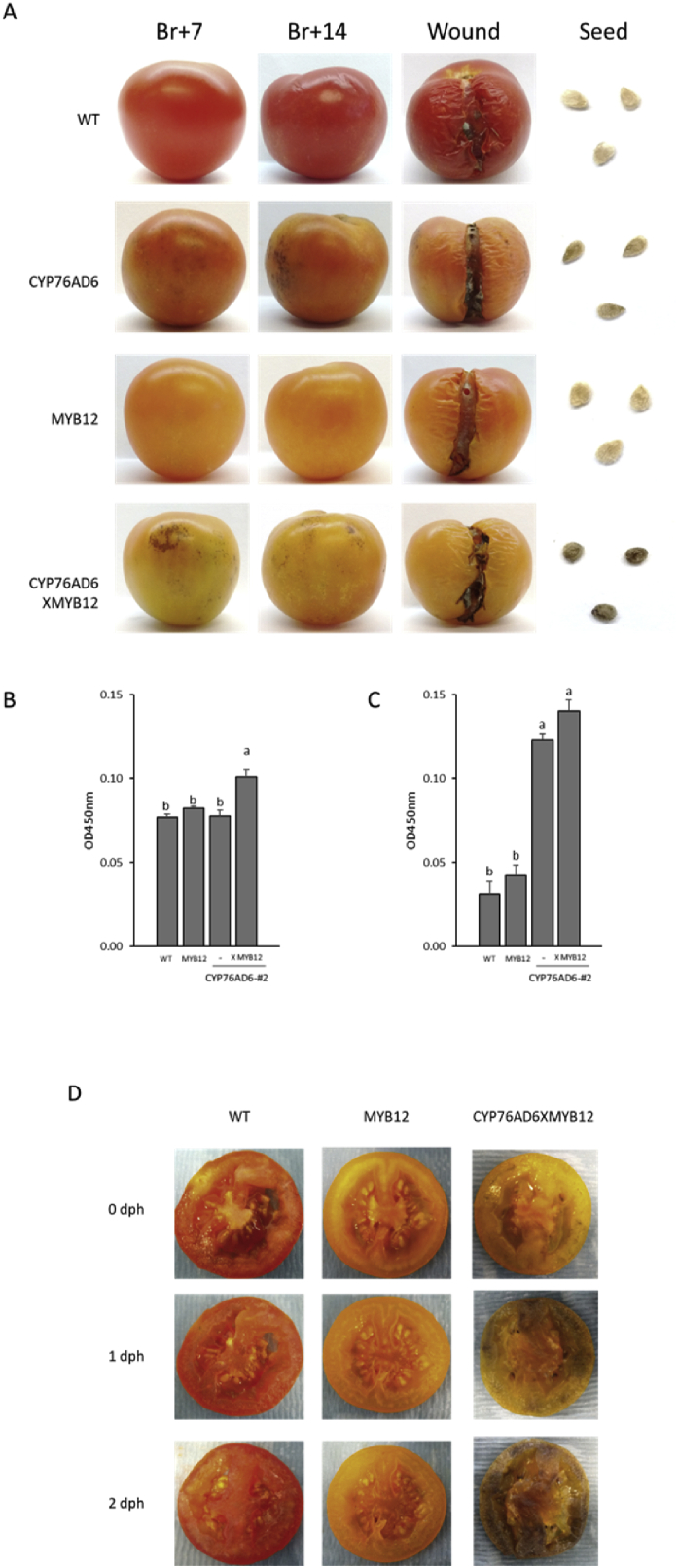
**Melanin accumulation in L-DOPA fruit:** (A) Fruit from wt, MYB12, *CYP76AD6*-expressing and CYP76AD6XMYB12 plants were harvested at 7 days post breaker (left column) and 14 days post breaker (middle column). Accumulation of the dark melanin-like pigment could be observed by 7 days post breaker in the L-DOPA-enriched fruit and at higher intensity in the fruit of CYP76AD6XMYB12 crosses. In addition, fruit were injured at 7 days post breaker and harvested at 14 days post breaker (right column). (B–C) Accumulation of melanin was evident in the CYP76AD6XMYB12 fruit at 14 days post breaker, in both (B) intact or (C) injured fruit, while in the *CYP76AD6*-expressing parental line, melanin levels were high only in injured fruit. (D) Sliced fruit, harvested at 7 days post breaker, exhibited rapid browning one day after treatment, which intensified further with time. Student's t-test significance: P-value<0.05; Days post harvest- dph.

Fruit expressing *CYP76AD6* were generally slightly darker than control fruits, and showed dark brownish spots on their surface, which were more prominent in the CYP76AD6XMYB12 fruit, and were not present in any of the control fruits (Fig. 7A-left and middle panels). We also noticed that when CYP76AD6-expressing fruit and CYP76AD6XMYB12 fruit were injured on the vine, particularly when fruit split, a dark pigment accumulated at the boundaries of the injured region (Supplemental Fig. S1). Oxidation of L-DOPA by PPOs can lead to the production of DOPA-quinone and, through further spontaneous polymerisation steps, to the formation of the dark pigment, melanin (Fig. 1B) (Hachinohe and Matsumoto, 2007; Kamkaen et al., 2007). We therefore assayed fruit 14 days post-breaker for melanin levels. Melanin accumulated to significant levels in the CYP76AD6XMYB12 fruits, which showed the highest levels of L-DOPA, although we could not be sure how much of this was produced by oxidation during extraction (Fig. 7B).

To investigate the browning in injured fruit, we made cuts on the fruit surface of fruit at 7 days post breaker and harvested seven days later. We found significant accumulation of melanin in cut fruit of both the L-DOPA-accumulating genotypes, and in those crossed to MYB12. This confirmed that the dark pigment was melanin and originated from the oxidation of L-DOPA (Fig. 7C). In addition, wt, MYB12 and CYP76AD6XMYB12 fruit harvested at seven days post breaker, were sliced and placed on humid paper on a sealed plate to examine the speed of browning. The CYP76AD6XMYB12 fruit showed browning the following day, which got stronger with time (Fig. 7D) establishing that oxidation of L-DOPA to melanin was low in intact fruit but occurred rapidly upon exposure to air.

## 4 Discussion

L-DOPA is a non-standard amino acid, and its importance in the medical field is well established for the treatment of the Parkinson's Disease (PD) (Hornykiewicz, 2010). In fungi, the enzymes that synthesise L-DOPA from tyrosine are PPOs called tyrosinases, which were identified as involved in the biosynthesis of melanin (Sato et al., 2009). However, in plants, CYP450 enzymes catalysing the production of L-DOPA from tyrosine were discovered only recently, from research on betalain biosynthesis, in which L-DOPA is an important intermediate (Sunnadeniya et al., 2016; Polturak et al., 2016; DeLoache et al., 2015). L-DOPA is generally not present at high levels in plants because it is consumed by different biosynthetic pathways, synthesising betalains, morphine, melanin, and other specialised metabolites (Soares et al., 2014; Polturak and Aharoni, 2018). A restricted number of plants accumulate L-DOPA, perhaps most notably the velvet bean, *Mucuna pruriens*, in its seeds (Ramya and Thaakur, 2007). Natural sources of L-DOPA are often used for the treatment of PD in cases where the patient suffers from adverse effects of chemically synthesised L-DOPA, such as nausea, vomiting and behavioural complications (Cummings, 1991; Barbeau, 1969).

The most widely studied natural source of L-DOPA is velvet bean (*Mucuna pruriens*) but this source is far from ideal, and problems arise from harvest through processing to its final applications. The plant is covered with urticating hairs that contain mucunain, that can cause irritation and allergic reactions in field workers that harvest the crop, and the beans themselves contain high levels of tryptamines, that can cause hallucinations in PD patients (Martín Ortega et al., 2019; Lampariello et al., 2012). In order to explore the possibilities of increasing the repertoire of plants accumulating L-DOPA, we introduced the enzyme responsible for converting tyrosine to L-DOPA in the betalain biosynthetic pathway into a non-producing plant, tomato, and produced L-DOPA-enriched tomato fruit. Tomato was the plant of choice, since it has been engineered to accumulate several other secondary metabolites which can reach high levels particularly when restricting production specifically to fruit, using the fruit-specific E8 promoter. Tomato has numerous additional resources that can be used to enhance L-DOPA levels, such as the fruit specific *MYB12* lines (Fu et al., 2018). Furthermore, since tomato is widely cultivated, this crop can be used for scale-up and potentially offers a standardised and controlled natural source of L-DOPA.

The engineered tomato fruit accumulated up to 0.15% L-DOPA as a proportion of the fruit dry weight (Fig. 5C). This almost doubled to 0.27% when *MYB12* was expressed ectopically in fruit to increase flux to tyrosine (Fig. 5A,C) (Pandey et al., 2015). These L-DOPA levels are similar to those accumulating in other non-seed organs of L-DOPA producing plants (Ramya and Thaakur, 2007). A common dose for L-DOPA treatment is less than 500 mg/day (Brooks, 2008). This dose could be achieved by about 200 g of dry matter (~2 kg fresh fruit) from our engineered tomato fruit. Furthermore, different sources of L-DOPA are widely accepted as traditional therapies for other purposes, such as male infertility and as an aphrodisiac, where lower doses of L-DOPA are consumed (Lampariello et al., 2012).

Interestingly, the ectopic accumulation of L-DOPA in tomato, revealed several accompanying effects, related to L-DOPA production in plants. Firstly, the shelf life properties of the fruit were improved, and this effect correlated with higher antioxidant capacities and reduced expression of genes involved in cell wall degradation, resulting in improved fruit firmness post-harvest, and reduced susceptibility to *B. cinerea* (Fig. 6). Synthesis of L-DOPA had major effects on the plant metabolome, including increased levels of both standard and non-standard amino acids in fruit (Fig. 3). Surprisingly, the levels of tyrosine itself were not significantly reduced, despite tyrosine being the precursor for L-DOPA, suggesting that metabolic regulation may be channelled to maintain tyrosine levels. This supports the reported tight regulation of tyrosine biosynthesis in plants through feedback inhibition (Lopez-Nieves et al., 2018). Tyrosine levels may be maintained by increases in the levels of intermediates in glycolysis and the TCA cycle. Reduced tocopherol levels indicated that metabolic flux had been shifted towards L-DOPA from other tyrosine-utilising pathways. This interpretation was confirmed by LC-MS analysis, which showed reduced levels of other nitrogen-containing and phenolic compounds in fruit accumulating L-DOPA (Fig. 4).

An interesting feature of the accumulation of L-DOPA was its spontaneous polymerisation to melanin. Following oxidation of L-DOPA to DOPA-quinone by PPOs, the generation of melanin does not require additional enzymes (Hachinohe and Matsumoto, 2007; Kamkaen et al., 2007). Indeed, we found that melanin was produced at low levels in L-DOPA-accumulating fruit and levels increased following exposure of L-DOPA-containing fruit tissue to air by injury (Fig. 7, Supplemental Fig. S1). Melanin synthesis has been associated with phytotoxicity through its inhibition of root elongation, lipid peroxidation and elevation of levels of reactive oxygen species (Hachinohe and Matsumoto, 2005, 2007). Melanin accumulation has been reported in Arabiodpsis plants expressing *BvCYP76AD5* (a *BvCYP76AD6* paralogue), and in lettuce treated with L-DOPA (Hachinohe and Matsumoto, 2007; Sunnadeniya et al., 2016). In the BvCYP76AD6 tomato plants any potential toxicity of L-DOPA itself or melanin was limited by restricting the accumulation of L-DOPA to ripe fruit, using the E8 fruit-specific promoter.

We have demonstrated that the use of the *CYP76AD6* expressing tomatoes as a source of L-DOPA is feasible and that they could be considered further as a source of L-DOPA for treatment of Parkinson's Disease in places where access to commercial pharmaceuticals is limited and/or relatively expensive. At estimated levels of 150 mg L-DOPA per kg tomatoes, the L-DOPA tomatoes yield much lower than microbial fermentation systems or immobilised tyrosinase bioproduction systems (Min et al., 2015). However, scale-up production of tomatoes is low-tech and high yields can be achieved without major investment (Zhang et al., 2013). In addition, we have shown that L-DOPA stores in the harvested tomatoes without major oxidation to melanin, provided the fruit are not wounded. The extended shelf-life of L-DOPA fruit could allow tomatoes to be harvested later than usual, with potentially further enhancement of L-DOPA content, because the E8 promoter switches on only at breaker during tomato fruit development (Butelli et al., 2008). Production of purified L-DOPA from these tomatoes could be achieved by homogenisation in water-methanol mixtures with added ascorbate to limit oxidation, followed by chromatographic separation such as thin layer chromatography (Vachhani et al., 2011). This would allow local, cheap, low-tech production of this important pharmaceutical for PD patients who currently lack access as a result of their location and/or its cost.

In addition, natural sources of L-DOPA, such as velvet been (which contains between 0.5 and 9% L-DOPA in its seeds), have been reported to show significantly better effects than commercially synthesised L-DOPA in the treatment of PD (Hussain and Manyam, 1997). The plant matrix is thought to play an important role in increasing the therapeutic effects of *Mucuna* extracts (Vaidya et al., 1979) and the antioxidant activity of extracts of velvet bean seeds can prevent the progress of oxidative stress (Lampariello et al., 2012; Dharmarajan et al., 2010). An increased antioxidant capacity resulting from accumulation of L-DOPA was also observed in *CYP76AD6*-expressing tomato fruit (Fig. 6), suggesting that natural extracts of these tomatoes (such as tomato water) could offer a substitute for velvet bean extracts used as an aphrodisiac or to treat male infertility.

## Declaration of competing interest

None.
