# Supplementary figures and images for: Metabolic engineering of tomato fruit enriched in L-DOPA
Source: Metab Eng. 2021 May;65:185–96. doi: 10.1016/j.ymben.2020.11.011 (PMC8054910; doi:10.1016/j.ymben.2020.11.011)

## Slide 1
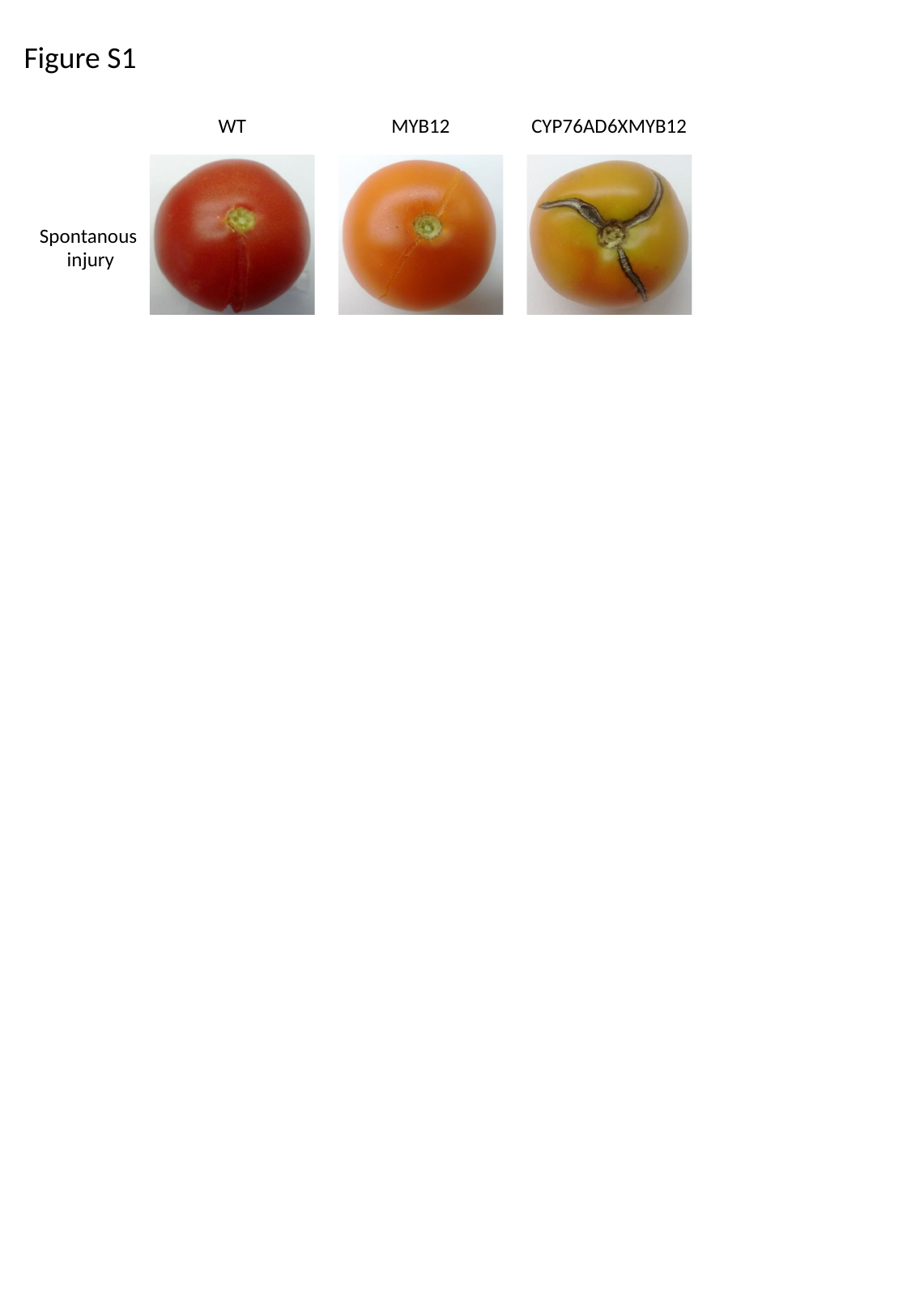

Figure S1
WT
MYB12
CYP76AD6XMYB12
Spontanous injury

Supplement: Multimedia component 1 [file mmc1.pptx]
